# Lupus Anticoagulant Testing for Diagnosis of Antiphospholipid Syndrome: A Perspective Informed by Local Practice

**DOI:** 10.3390/jcm14144812

**Published:** 2025-07-08

**Authors:** Emmanuel J. Favaloro, Leonardo Pasalic

**Affiliations:** 1 Haematology, Sydney Centres for Thrombosis and Haemostasis, Institute of Clinical Pathology and Medical Research (ICPMR), NSW Health Pathology, Westmead Hospital, Westmead, NSW 2145, Australia; leonardo.pasalic@health.nsw.gov.au; 2 Sydney Centres for Thrombosis and Haemostasis, Research and Education Network, Westmead Hospital, Westmead, NSW 2145, Australia; 3 School of Dentistry and Medical Sciences, Faculty of Science and Health, Charles Sturt University, Wagga Wagga, NSW 2650, Australia; 4 School of Medical Sciences, Faculty of Medicine and Health, University of Sydney, Westmead Hospital, Westmead, NSW 2145, Australia; 5 Westmead Clinical School, University of Sydney, Westmead, NSW 2145, Australia

**Keywords:** antiphospholipid syndrome, lupus anticoagulant, activated partial thromboplastin time, dilute Russell viper venom time, anticoagulant therapy

## Abstract

Assessment for the presence or absence of lupus anticoagulant (LA) represents a common investigation in hemostasis laboratories. In particular, LA represents one of the laboratory criteria for the diagnosis of definite antiphospholipid syndrome (APS). The other laboratory criteria are the solid phase assays (anticardiolipin (aCL) and anti-β2Glycoprotein I (aβ2GPI) antibodies of IgG and IgM isotypes). Current International Society on Thrombosis and Haemostasis (ISTH) guidance recommends testing LA by at least two tests based on different principles, with the activated partial thromboplastin time (aPTT) and dilute Russell viper venom time (dRVVT) being preferred. Additional assays may be used in addition, or instead of these assays in particular situations. For example, aPTT and dRVVT assays are very sensitive to the presence of various anticoagulants, and this may lead to false-positive identification of LA. This is particularly problematic in the age of the DOACs (direct oral anticoagulants), which are now the leading anticoagulants in use worldwide. We review recent literature on LA testing as well as our local practice to provide an update on this common test procedure. Our experience should be useful for laboratories struggling with LA interpretation for diagnosis or exclusion of APS.

## 1. Introduction

Lupus anticoagulant (LA) represents one of the laboratory criteria for the diagnosis of definite antiphospholipid syndrome (APS) [1]. LA as a laboratory classification criterion has stood the test of time, with inclusion continuing on from past versions of APS classification documents [2,3]. The other laboratory classification criteria are the solid phase assays for anticardiolipin (aCL) and anti-β2Glycoprotein I (aβ2GPI) antibodies of IgG and IgM isotypes [1,2,3]. The presence of at least one of these laboratory criteria, together with at least one clinical criterion, defines definite APS for inclusion of characterized patients in clinical trials and for publication of APS research. The main clinical criteria are thrombosis and pregnancy/fetal morbidity, but several other clinical criteria are also listed [1,2,3]. The classification criteria are often also used by clinicians, usually inappropriately, as APS diagnostic criteria; however, the authors of the classification criteria [1] and others [4,5,6,7] explain that the classification criteria represent a narrow range of laboratory and clinical criteria, and that a much broader range of laboratory tests and clinical pathology can define APS diagnostically.

Results from antiphospholipid antibody assays are similarly used within the classification criteria of Systemic Lupus Erythematosus (SLE) [8], as well as for its diagnosis and disease profiling [9,10,11].

**Figure 1:**
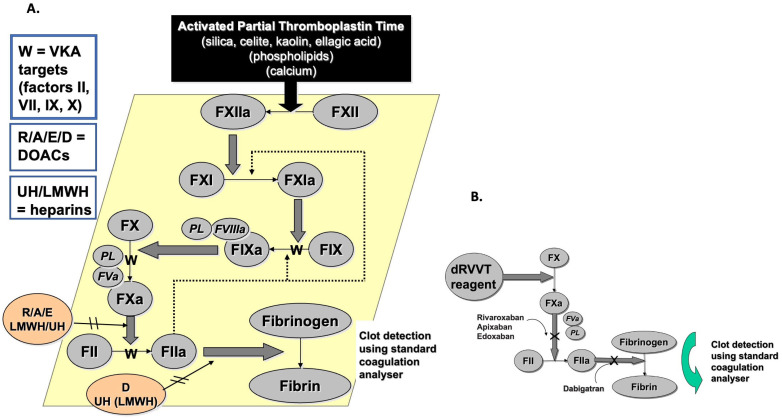
A representation of the activated partial thromboplastin time (aPTT; (**A**)) and the dilute Russell viper venom time (dRVVT; (**B**)). Both assays can be made LA-sensitive by inclusion of a limited amount of phospholipid (PL), to which the LA antibodies can bind and functionally inhibit clotting, thereby prolonging test times. These LA-sensitive assays are used as LA screening tests. Both assay types can also be made relatively LA-insensitive by inclusion of excess amounts of phospholipid (PL), which is then able to swamp the LA antibodies and thus generate normal or less prolonged test times compared to the LA-sensitive versions. These relatively LA-insensitive assays are used as LA confirmation tests. All aPTT and dRVVT test types are variously affected by various clinical anticoagulants that can interfere with clotting and prolong test times, and in some cases can even mimic LA, thereby leading to false-positive LA. Abbreviations: A, apixaban; D, dabigatran; DOAC, direct oral anticoagulant; E, edoxaban; F, factor; LMWH, low molecular weight heparin; PL, phospholipid; UH, unfractionated heparin; VKA, vitamin K antagonist; W, warfarin.

The term ‘lupus anticoagulant’ is considered to be a double misnomer, as it neither represents an anticoagulant nor is it exclusively associated with lupus disease. Originally identified as associated with lupus disease [12,13], LA represents antibodies that bind to phospholipids (PL), typically in association with various cofactor proteins, and which act to inhibit PL-dependent clotting assays such as aPTT and dRVVT (Figure 1), thereby prolonging these LA-sensitive assays.

LA evaluations can be undertaken in a number of ways. However, the latest International Society on Thrombosis and Haemostasis (ISTH) guidance [5] also continues the tradition of past ISTH guidance documents [7,14,15] to recommend performance of at least two clot-based tests based on different principles before excluding LA. The most frequent and highly recommended tests comprise an LA-sensitive aPTT (activated partial thromboplastin time) and an LA-sensitive dRVVT (dilute Russell Venom Time) [5,7,14,15] (Figure 1). To diagnose LA, only one of these needs to be identified as being positive for LA. The basic tenant of the laboratory test process has also stood the test of time [5,7,14,15], and comprises three steps: 1. prolongation of at least one of these LA-sensitive tests (also called ‘screening assays’), 2. evidence of an inhibitor-like pattern with mixing studies, and 3. significant evidence of shortening or normalization of the clotting tests using paired reagents for aPTT and dRVVT that are relatively insensitive to LA (also called ‘confirmation assays’).

In some situations, additional or alternate tests can be recommended or performed [5,16,17,18,19]. For example, although several manufacturers provide paired dRVVT screening (i.e., LA-sensitive) and confirmation (i.e., relatively LA-insensitive) tests, there are very few truly paired aPTT assays that share these characteristics [20]. Thus, one possible alternative to a paired aPTT reagent panel is a paired silica clotting time (SCT) assay, since these reflect a similar test principle (contact pathway) to the aPTT [5]. Also, since clinical anticoagulants affect clotting assays, and many patients under investigation for APS are under anticoagulation therapy, some relatively anticoagulant-insensitive assays can be used instead of aPTT and dRVVT. For example, one possible pairing is the Taipan snake venom time/ecarin time (TSVT/ET) combination. These tests are relatively insensitive to anti-Xa anticoagulants, and thus are proposed to identify LA in certain settings [5,18,19]. These tests also exhibit high sensitivity to LA in triple aPL-positive nonanticoagulated patients [18,19].

## 2. The Basic LA Diagnosis/Exclusion Process

**Figure 2:**
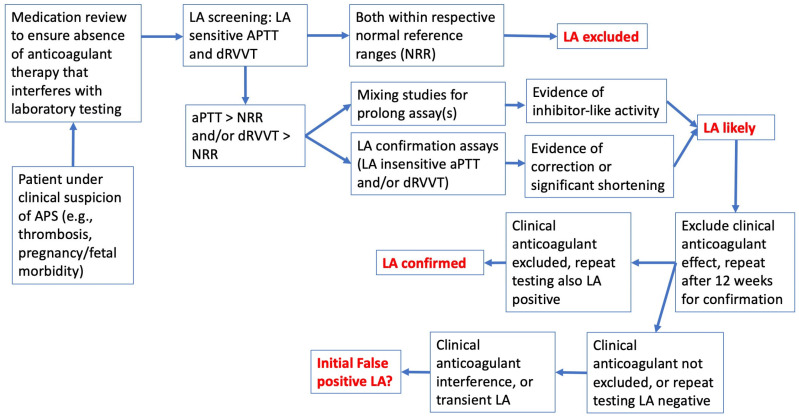
The basic process for exclusion or diagnosis of lupus anticoagulant (LA) “Part 1”. The first step is to assess patient’s clinical history, especially for pathology related to antiphospholipid syndrome (APS) such as thrombosis and pregnancy/fetal morbidity, as well as medication use. Then, if anticoagulation therapy affecting clotting assays is excluded, patient plasma is assessed for LA using at least two LA screening assays based on different principles (such as the aPTT and dRVVT). If both assays generate test results within their normal reference range (NRR), then LA can be excluded. If either assay yields a prolonged test time, then LA is feasible and this requires further steps, including mixing studies and additional testing with relatively LA-insensitive (LA “confirmation” assays) based on the same assay principles as the screening tests. As clinical anticoagulants may also interfere with these tests (Figure 1), these should be excluded as causes of test prolongation, before diagnosing LA. In particular, all DOACs can lead to false-positive LA, while apixaban can lead to false-negative LA in some cases. LA should also be confirmed by repeat testing after 12 weeks to exclude transient LA.

There are many challenges to the diagnosis of APS, and indeed even LA [5,21,22]. The first step in assessing patients for APS for the clinician is to assess patient’s clinical history, especially for pathology related to APS. This will include evident thrombosis and pregnancy/fetal morbidity, as well as many additional clinical signs [23,24,25,26,27,28,29]. In addition, it is important to note if the patient is on any clinical anticoagulants for their condition, since anticoagulants will affect most clotting tests, inclusive of those used for LA diagnosis/exclusion [30,31,32]. Then, patient blood is collected from the patient, and serum is assessed for solid phase assays for aCL and aβ2GPI, and plasma is assessed for LA using at least two LA screening assays based on different principles [5]. In our laboratory, this would be the aPTT and dRVVT for LA testing. If both assays generate test results within their respective normal reference range (NRR), then LA can be excluded [5] (Figure 2). If either assay yields a prolonged test time, then LA is feasible, as are other conditions, and thus LA diagnosis/exclusion requires further steps, including mixing studies and reflex testing with relatively LA-insensitive confirmation assays based on the same assay principles as the screening tests [5]. As clinical anticoagulants can interfere with these tests (Figure 1, Table 1) [30,31,32,33], these anticoagulants should also be excluded as possible causes of test prolongation. LA should also be confirmed by repeat testing after 12 weeks to exclude transient LA [5], such as that associated with infections, including that causing COVID-19 (coronavirus disease 2019) [34].

As noted above, LA can be excluded if two tests, based on different principles, and otherwise sensitive to LA, do not yield abnormal test times with patient plasma (i.e., patient is LA “negative”; Figure 2). To identify LA presence, either or both of these two tests should be LA “positive”, meaning initial prolongation in the screening assay, inhibitor-like activity in mixing studies, and correction or significant shortening in confirmation assays [5]. In the past, this three-step process was performed sequentially. In the latest guidance [5], the last two steps are recommended to be done at the same time.

It is very important to exclude the possibility of clinical anticoagulants, since these may interfere with LA tests (Figure 1), and also lead to test prolongation. In some cases, test patterns using plasma from anticoagulated patients can mimic the presence of LA [30,31,32,33]. In particular, the direct oral anticoagulants (DOACs) pose the biggest threat for false-positive/false-negative LA. Moreover, the dRVVT is very sensitive to all the DOACs. Some DOACs (e.g., dabigatran and rivaroxaban) affect the dRVVT screening assays more than LA confirmation assays, leading to falsely raised screen/confirmation ratios, which are often used as surrogates for LA diagnosis [30,31,32,33,35].

## 3. True Positive LA vs. False-Positive LA vs. Anticoagulant Interference

As noted, many clinical anticoagulants will interfere with the assays used to assess LA [30,31,32,33]. This is relevant since anticoagulant therapy is one of the major treatments for patients at risk of thrombosis, or under treatment for a recent thrombosis. For example, heparin is a parenteral agent often used within the hospital system, and available as ‘unfractionated heparin’ (UH) or as ‘low molecular weight heparin’ (LMWH). These heparins, especially UH, will interfere with both aPTT and dRVVT assays (Figure 1), as well as silica clotting tests (SCT), which are sometimes used in place of the aPTT for LA investigation. To counteract this, most commercial dRVVT reagents contain a heparin neutralizer able to eliminate interference from therapeutic levels of heparin. However, most aPTT and SCT reagents do not contain these neutralizers, and indeed, the aPTT is often used to help monitor UH therapy [36]. Vitamin K antagonists (VKA) lead to the production of dysfunctional vitamin K-dependent factors (i.e., FII, FVII, FIX, FX) and thus VKA therapy will also lead to prolongations in all three assays (Table 1). Finally, the newest class of clinical anticoagulants, the so-called DOACs, specifically inhibit either factor IIa (e.g., dabigatran) or factor Xa (e.g., apixaban, rivaroxaban, edoxaban). DOACs will also lead to prolongations in both aPTT and dRVVT assays, but also in some cases will mimic LA activity [30,31,32,33].

There are various strategies that can be used to mitigate anticoagulant interference in LA testing (Table 1). As noted, most commercial dRVVT reagents already contain heparin neutralizers to eliminate heparin interference. Whilst aPTT and SCT reagents may not contain heparin neutralizers to eliminate heparin interference, one strategy could be to replace the existing CaCl_2_ reagents with a CaCl_2_ reagent containing a heparin neutralizer [30]. Alternatively, patients can be transitioned from UH to LMWH prior to LA investigation, since LMWH expresses far less assay interference [5,37,38].

For VKA therapy, the simplest mitigation strategy is to perform LA testing on 1:1 patient plasma/normal plasma mixes [5]. Mixing patient plasma with normal plasma overcomes the vitamin K-dependent factor deficiency. However, some workers feel this strategy may yield both false-positive and false-negative LA findings [5,37].

However, DOACs currently pose the greatest risk in LA testing. DOACs are now very widely used anticoagulants/antithrombotics [39], and this class of clinical anticoagulants causes the greatest assay interference in LA testing [30,31,32,33]. Indeed, both rivaroxaban and dabigatran can lead to false-positive LA test patterns. This is since these drugs affect screening assays more than confirmation assays, yielding falsely elevated screen/confirm ratios. Indeed, in one multi-laboratory assessment of test practice, a rivaroxaban-containing sample was falsely identified as an LA-positive sample by the majority of study participants [40]. In contrast, apixaban can lead to false-positive or false-negative LA test patterns [30,33].

Although commercial aPTT, SCT, and dRVVT test reagents do not contain DOAC neutralizers, there are strategies to remove DOAC-related assay interference. One successful strategy is the pretreatment of DOAC-containing plasma with a commercial DOAC neutralizer, such as DOAC Stop [30,35,36]. These products contain activated charcoal that will absorb DOACs from the plasma, leaving a DOAC-free plasma for LA testing. However, once added to plasma, the resulting mixture is very turbid, and will itself also interfere with the detection of clot formation. Therefore, these products usually need to be removed by centrifugation prior to testing plasma in LA tests [41]. This is especially true for optical detection methods, but may be perhaps less so for mechanical test systems [41]. Of course, another mitigation strategy would be to cease DOAC therapy for 48–72 h or to transition patients temporarily to LMWH therapy ahead of LA testing [5,37,38]; however, clinicians may be reluctant to do this.

In any case, it should now be very clear why patient anticoagulant status must be disclosed by the clinician to the LA testing laboratory, so that they can undertake the appropriate mitigation strategy.

## 4. Interpretation and Reporting of Test Results—The Westmead Approach

**Figure 3:**
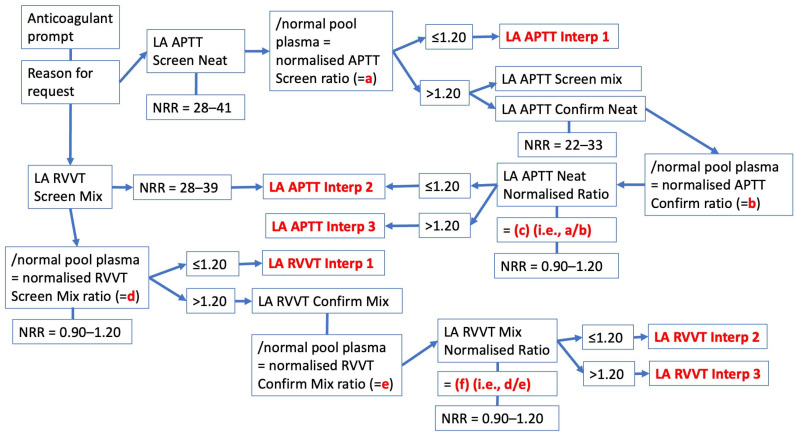
The basic process for exclusion or diagnosis of lupus anticoagulant (LA) “Part 2”. This algorithm is an extension of that shown in Figure 2. See Table 2 for full interpretive comments associated with individual tests (aPTT and dRVVT).

Different laboratories may report results for LA in many different ways [5]. Testing for LA comprises several test components. The simplest test result outcome is where LA is not detected (or LA is ‘negative’). In this situation, the laboratory will, in general, only need to perform two tests based on different principles. In our laboratory, this will be the aPTT using Cephen reagents (Hyphen BioMed, Neuville-sur-Oise, France), and dRVVT using Werfen reagents (Barcelona, Spain), but only using the screening assays (Figure 2 and Figure 3) [42]. Our laboratory also uses normalized test ratios to adjust for day to day and reagent lot variations; this is simply the test time obtained from the patient for the specific assay divided by the test time obtained with a pool of normal plasma (PNP; we just use a commercial pool plasma) for the same assay. Most of our normalized ratios yield a normal reference range of around 0.90–1.20 [42]. In our laboratory, the process has been fully automated within our instrumentation (Werfen ACL-TOP 750; Barcelona, Spain) and laboratory information system (LIS) using an algorithmic approach. The basic algorithmic approach process undertaken is shown in Figure 2 and Figure 3.

In order to request an LA investigation from our laboratory, the clinician is first prompted to identify any clinically relevant patient anticoagulant, as well as provide a clinical reason for the investigation (e.g., thrombosis, pregnancy morbidity) (Figure 2, Figure 3). Once the blood sample is collected, and processed to generate double-spun plasma as per ISTH guidance [5], both aPTT and dRVVT screening assays are then performed. These tests are reported in seconds, relative to the test NRR, and are also automatically calculated as normalized ratios based on the pool normal plasma result from the same assay test run (Figure 3). Our laboratory initially performs the aPTT screen using neat patient plasma, and performs the dRVVT assay as a 1:1 patient/PNP mixed plasma dRVVT screen. The latter approach is our historical approach, since we determined that many of our past patient samples derived from patients on anticoagulant therapy, historically VKAs, and using the mix plasma step overcomes VKA interference. Although some patients are still on VKA therapy, the majority are now on DOAC therapy [39,43]. Nevertheless, performing a mixed plasma dRVVT screening helps to dilute or reduce the DOAC interference [33,40]. Using the mix plasma test also in part satisfies the requirement to identify an inhibitor-like activity [5]. If both aPTT and dRVVT screens and respective normalized ratios are within the NRRs, LA is said to be excluded, and the process automatically generates individual test interpretations (LA APTT Interp 1 and LA dRVVT Interp 1) (See also Table 2). If either aPTT or dRVVT test results or their normalized ratios exceed their NRR, then the process continues as shown in Figure 3, including performance of additional (confirmatory) tests, and different individual test interpretations are generated (LA APTT Interp 2 or LA APTT Interp 3, and LA dRVVT Interp 2 or LA dRVVT Interp 3) (see also Table 2). Finally, the automated process combines these individual test possibilities into a final LA interpretation based on the possible combination of individual test interpretations (Table 3).

## 5. Discussion

In this perspective article we have reviewed the diagnosis or exclusion of LA as a marker of APS, as well as providing perspectives of laboratory reporting and interpretation based on our local test practice. This perspective has in part been informed by ISTH guidance, both current and past [5,14,15], with one of us being a member of the ISTH committee in charge of this guidance [5,14,15]. This perspective has also been in part informed by our recent large scale multicenter evaluation of LA testing within our large pathology network [42], our work on LA anticoagulant interference and strategies to overcome this interference [33,38,39,40,41], and a long history of involvement in hemostasis practice [44].

In this respect, however, it is also important to consider the limitations of our experience. For example, we have no experience with the combination of Taipan snake venom time (TSVT) with ecarin time (ET) for investigation of LA, and so for these tests we would point readers to other experts in their use [18,19,45]. Our own historical strategy of using mixing tests to overcome historical VKA therapy interference in LA testing has been retained, but perhaps has less relevance in 2025 since VKA therapy has undergone considerable reduction in the last decade [39]. Nevertheless, it still retains relevance, because VKA therapy is still in place for a subset of APS patients, especially those with more severe disease (e.g., triple-positive APS) [43,46,47]. The strategy also helps to reduce LA test interference in patients on DOAC therapy [30,40].

It is also important for readers to note that not all LA detected by the laboratory has clinical consequences. LA investigations are sometimes carried out in asymptomatic patients simply because the laboratory has identified a prolonged aPTT test in some coagulation screening process investigating a patient for some possible hemostasis disorder, perhaps ahead of intended surgery. Most aPTT reagents are purposely designed to be sensitive to factor deficiencies (for investigation of potential bleeding defects), as well as UH (for monitoring of UH) [36]. In addition, more aPTT reagents are sensitive to LA than are not sensitive to LA [20], and indeed, the number of relatively LA-insensitive aPTT reagents for use as LA confirmation assays remains low [20]. Nevertheless, guidance from the ISTH [5,7,14] and others [48,49], recommends the use of a relatively LA-insensitive aPTT reagent for screening of hemostasis to avoid detection of asymptomatic LA with aPTT screening. For example, Martini et al. recently published on this situation in children being screened by the aPTT ahead of planned surgery [50]. They advised that a prolonged preoperative aPTT in children is often the cause of a delay of scheduled surgeries and the repetition of multiple blood tests, with the consequent wasting of resources and significant discomfort for children and parents. They also provided readers with some keys to interpret this situation and the possibility to correctly evaluate the hemorrhagic risk of a patient.

**Figure 4:**
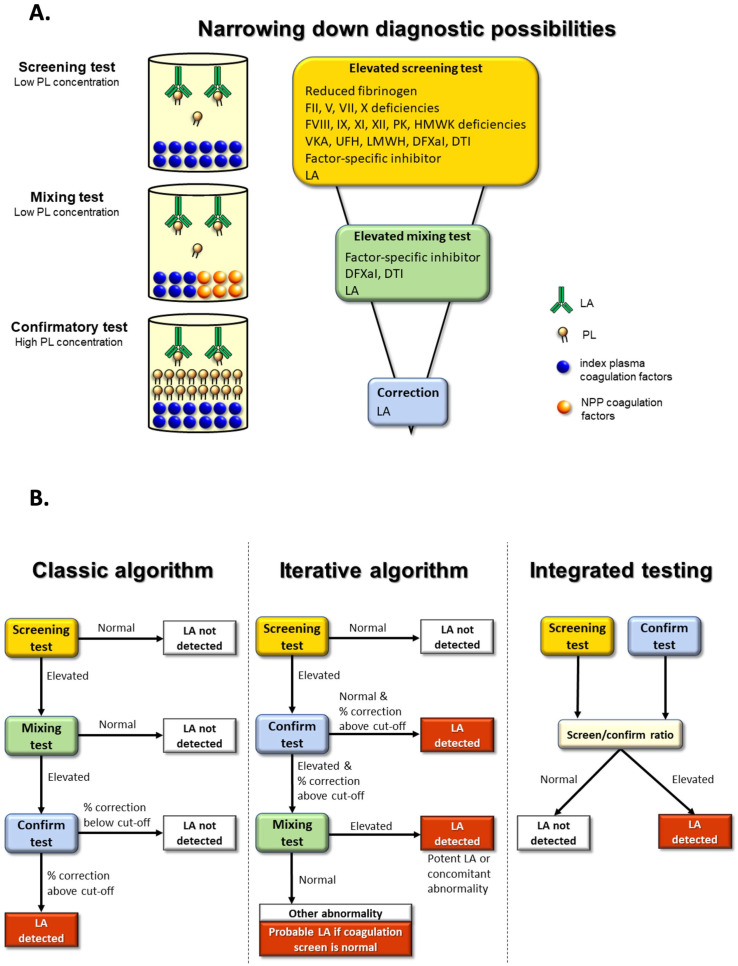
(**A**) A simplified algorithmic approach to diagnosis or exclusion of LA, from Moore (2022) [51], used with the permission of the publisher. (**A**) The basic tenet of LA investigation with the three-step procedure for lupus anticoagulant detection acts to narrow the possibilities. (**B**) Lupus anticoagulant detection algorithms. The classic algorithm is a strict exclusion process, while the iterative algorithm permits interpretation based on knowledge of assay limitations. Integrated testing is convenient and works well in uncomplicated cases, but it oversimplifies complex cases. It can be improved by adding a mixing step for complex cases.

Also, just as our algorithmic approach is based on our experience, there are, of course, many other different approaches. For example, Moore, an international expert on LA, provides his own more simplified algorithmic approach to LA exclusion/detection in a recent review [51], as replicated in Figure 4. Additional guidance and potentially alternate perspectives have been provided by other authors recently publishing in this journal [52,53,54,55]. For example, from our perspective, we have focused on LA and clotting tests. Tohidi-Esfahani et al. instead focus their report on platelets and APS [55]. In particular, platelets have established roles in thrombosis that may occur at any vascular site, and platelet hyperreactivity is clearly demonstrated in the pathophysiology of APS [55]. Of interest, despite the potential role of platelets in thrombosis, including that arising from APS, antiplatelet therapies only play a minor role in APS treatment, potentially reserved as a possible option as low-dose aspirin in addition to VKA, in arterial or refractory thrombosis.

Lastly, LA is but one aPL class investigated in APS. LA is detected or excluded based on clotting tests, usually performed within specialized hemostasis laboratories such as ours, and requiring patient plasma. A separate class of solid-phase immunologically based aPL assays is also performed for investigation of APS, usually within immunology laboratories using serum [5]. In our own situation, our laboratory also undertakes aPL testing with these solid phase assays, namely aCL and aβ2GPI, and these days specifically by a chemiluminescence assay on our Werfen (Barcelona, Spain) AcuStar analyzer [56]. A different set of strategies is required to optimize these tests for APS. Guidance is again provided by various experts, including from the ISTH [5,25,26,47,49,54]. Moreover, there are several other assays under evaluation for potential utility in APS diagnosis [5,16,57].

## 6. Conclusions

LA evaluation is complex, and there are many possible options for tests and procedures. We outline our own approach, as informed by a long history of activity in this field. We take into consideration clinical anticoagulation, as this may cause test interference, and potentially different strategies for eliminating this interference. Finally, even anticoagulation therapy is a moving landscape, and although DOACs are the current dominant anticoagulants in use worldwide, as other anticoagulants are developed, this may not always hold true [58], and thus a refresh of perspectives may also be required in time.

## Figures and Tables

**Table 1:** Anticoagulant interference in LA testing. Anticoagulants prolong most coagulation tests, including those used for LA investigation.

Anticoagulant	APTT	dRVVT	SCT
Unfractionated heparin	**↑**–**↑↑↑** (concentration-dependent; most reagents do not contain neutralizer)	**⟷** (up to ~1 U/mL heparin neutralized if reagent contains heparin neutralizer) **↑** (if exceeds neutralizer capacity)	**⟷** (up to ~1 U/mL heparin neutralized if reagent contains heparin neutralizer) **↑** (if exceeds neutralizer capacity)
LMWH	**↑**	**⟷** (if contains heparin neutralizer)	**⟷** (if contains heparin neutralizer) **↑** (if no neutralizer)
VKAs	**↑**	**↑↑**	**↑**
dabigatran	**↑↑**	**↑↑↑**	**↑**
rivaroxaban	**↑**	**↑↑↑**	**↑↑**
edoxaban	**↑**	**↑↑**	**↑↑**
apixaban	**⟷**–**↑** (assay-dependent)	**↑** (but LA ratio may fall, since effect greater on confirm reagents)	**↑**

aPTT, activated partial thromboplastin time; dRVVT, dilute Russell viper venom time; LMWH, low molecular weight heparin; SCT, silica clotting time; VKAs, vitamin K antagonists such as warfarin. ⟷, no change; ↑, increase.

**Table 2:** Test interpretations associated to single tests (i.e., aPTT or dRVVT), using our algorithmic approach previously shown in Figure 3.

Individual Test Interp	Interpretive Comment
LA APTT Interp 1	Lupus anticoagulant (lupus inhibitor) not detected by APTT method
LA APTT Interp 2	Lupus anticoagulant (lupus inhibitor) test performed by APTT method, with result not detected. However, screening test suggests potential presence of other inhibitor type
LA APTT Interp 3	Lupus anticoagulant (lupus inhibitor) test performed by APTT method, with positive detected (LA APTT ratio > 1.2)
LA RVVT Interp 1	Lupus anticoagulant (lupus inhibitor) not detected by dRVVT method
LA RVVT Interp 2	Lupus anticoagulant (lupus inhibitor) test performed by dRVVT method, with result not detected. However, screening test suggests potential presence of other inhibitor type
LA RVVT Interp 3	Lupus anticoagulant (lupus inhibitor) test performed by dRVVT method, with positive detected (LA dRVVT ratio > 1.2)

**Table 3:** A summary of the interpretations generated from various combinations of potential outcomes of individual tests (aPTT or dRVVT), using our algorithmic approach previously shown in Figure 3, and the individual test interpretations shown in Table 2.

LA APTT	LA dRVVT	Final LA Interpretive Comments
Interp 1	Interp 1	Lupus anticoagulant (lupus inhibitor) not detected by APTT & dRVVT methods.
Interp 1	Interp 2	Lupus anticoagulant (lupus inhibitor) not detected by APTT & dRVVT methods. However, dRVVT screening test suggests potential presence of other inhibitor type. If patient on anticoagulant therapy (vitamin K antagonist, heparin, or a direct oral anticoagulant [dabigatran, rivaroxaban, apixaban]), please repeat testing when therapy ceased. Otherwise, please discuss with laboratory as further testing may be required.
Interp 1	Interp 3	Lupus anticoagulant (lupus inhibitor) test detected by dRVVT method, but not APTT method. If patient on anticoagulant therapy (vitamin K antagonist, heparin, or a direct oral anticoagulant [dabigatran, rivaroxaban, apixaban]), please repeat test when therapy ceased, as test result may not be reliable and may reflect a false-positive. Otherwise, suggest repeat testing after at least 12 weeks for confirmation.
Interp 2	Interp 1	Lupus anticoagulant (lupus inhibitor) not detected by APTT & dRVVT methods.
Interp 2	Interp 2	Lupus anticoagulant (lupus inhibitor) not detected by APTT & dRVVT methods. However, APTT and dRVVT screening tests suggest potential presence of other inhibitor type. If patient on anticoagulant therapy (vitamin K antagonist, heparin, or a direct oral anticoagulant [dabigatran, rivaroxaban, apixaban]), please repeat testing when therapy ceased. Otherwise, please discuss with laboratory as further testing may be required.
Interp 2	Interp 3	Lupus anticoagulant (lupus inhibitor) test detected by dRVVT method, but not APTT method. APTT screening test suggests potential presence of other inhibitor type. If patient on anticoagulant therapy (vitamin K antagonist, heparin, or a direct oral anticoagulant [dabigatran, rivaroxaban, apixaban]), please repeat test when therapy ceased, as test result may not be reliable and may reflect a false-positive. Also assess test results with solid phase antiphospholipid antibody tests aCL and aβ2GPI to identify possible double or triple positivity. Otherwise, suggest repeat testing after at least 12 weeks for confirmation.
Interp 3	Interp 1	Lupus anticoagulant (lupus inhibitor) test detected by APTT method, but not dRVVT method. If patient on anticoagulant therapy (vitamin K antagonist, heparin, or a direct oral anticoagulant [dabigatran, rivaroxaban, apixaban]), please repeat test when therapy ceased, as test result may not be reliable and may reflect a false-positive. Also assess test results with solid phase antiphospholipid antibody tests aCL and aβ2GPI to identify possible double or triple positivity. Otherwise, suggest repeat testing after at least 12 weeks for confirmation.
Interp 3	Interp 2	Lupus anticoagulant (lupus inhibitor) test detected by APTT method, but not dRVVT method. dRVVT screening test suggests potential presence of other inhibitor type. If patient on anticoagulant therapy (vitamin K antagonist, heparin, or a direct oral anticoagulant [dabigatran, rivaroxaban, apixaban]), please repeat test when therapy ceased, as test result may not be reliable and may reflect a false-positive. Also assess test results with solid phase antiphospholipid antibody tests aCL and aβ2GPI to identify possible double or triple positivity. Otherwise, suggest repeat testing after at least 12 weeks for confirmation.
Interp 3	Interp 3	Lupus anticoagulant (lupus inhibitor) test detected by both APTT and dRVVT methods. If patient on anticoagulant therapy (vitamin K antagonist, heparin, or a direct oral anticoagulant [dabigatran, rivaroxaban, apixaban]), please repeat test when therapy ceased, as test result may not be reliable and may reflect a false-positive. Also assess test results with solid phase antiphospholipid antibody tests aCL and aβ2GPI to identify possible double or triple positivity. Otherwise, suggest repeat testing after at least 12 weeks for confirmation

## Data Availability

Data supporting results reported in this manuscript can be found in the public domain, as identified in the reference list.
